# High social support is associated with reduced cardiac events in patients following ICD/CRT-D implantation: a one-year follow-up study in China

**DOI:** 10.1186/s40359-025-03912-5

**Published:** 2025-12-30

**Authors:** Pan Jing, Shaoshan Pang, Lirui Yu, Si Lu, Hongyan Cai, Zhao Hu, Min Zhang

**Affiliations:** 1 Cardiology Department, the First Affiliated Hospital of Kunming Medical University, 295# Xichang Road, Kunming, 650032 Yunnan China; 2 Geriatric Cardiology Department, the First Affiliated Hospital of Kunming Medical University, 295# Xichang Road, Kunming, 650032 Yunnan China

**Keywords:** Social support, post-ICD/CRT-D implantation, Anxiety, Depression, Composite cardiovascular outcomes

## Abstract

**Background:**

Few studies exist on the impact of social support on cardiac events following implantation of implantable cardioverter-defibrillators (ICD) or cardiac resynchronization therapy devices with defibrillators (CRT-D). This study aims to investigate the longitudinal association between social support and adverse cardiac events among ICD/CRT-D recipients during a 1-year follow-up.

**Methods:**

This hospital-based longitudinal study included 101 patients undergoing first ICD/CRT-D implantation. Demographic and clinical data were collected as confounders. Social support, anxiety, and depression scores were assessed at baseline and during follow-ups (1, 3, 6, 12 months). Cardiac events (primary endpoint: all-cause mortality or appropriate ICD shocks; secondary endpoint: primary endpoint events, heart failure hospitalization, or acute coronary syndrome) were documented. Kaplan-Meier analyses and Cox proportional hazards models (adjusted for age, sex, NYHA class, and ICD indication) evaluated associations, while time-dependent Cox models analyzed temporal effects.

**Results:**

Patients with higher baseline social support exhibited a reduced risk of the primary composite endpoint (log-rank χ² = 5.53, *p* = 0.019) and secondary composite endpoint (log-rank χ² = 14.64, *p* < 0.001). After multivariable adjustment (age, sex, NYHA class, ICD indication, anxiety/depression scores), high social support was independently linked to 65.6% lower secondary endpoint risk (adjusted HR = 0.344, 95% CI: 0.172–0.688, *p* = 0.003). The protective association attenuated over time (HR = 0.991 per unit time, *p* = 0.009) but remained statistically significant. Anxiety and depression showed no baseline association with events but demonstrated a time-dependent risk increase.

**Conclusion:**

Higher social support is independently associated with a reduced risk of the secondary composite endpoint after ICD/CRT-D implantation. Sustained psychosocial interventions may be warranted to mitigate long-term risk attenuation.

**Supplementary Information:**

The online version contains supplementary material available at 10.1186/s40359-025-03912-5.

## Introduction

Sudden cardiac death (SCD) refers to an unexpected death from a cardiac cause occurring within one hour after the onset of acute symptoms [1]. Malignant ventricular arrhythmias are reported to be the most common cause of SCD, as over 80% of SCD is caused by malignant ventricular arrhythmias [2]. Clinical trials have shown that implantable cardioverter-defibrillator (ICD) and cardiac resynchronization therapy with defibrillator (CRT-D) can reduce SCD incidence and all-cause mortality in high-risk individuals, making it the preferred prevention method for those with heart diseases [3–6]. A European multicenter study found that nearly 50% of patients experienced improved quality of life (QoL) after ICD/CRT-D implantation, especially with CRT-D [7]. However, in some patients with ICD/CRT-D, their improved QoL may be attenuated by symptoms of psychosocial factors, such as anxiety or depression, when a shock is necessary to accomplish cardioversion or defibrillation [8]. Studies indicated that anxiety and depression severely reduce the QoL for patients with ICD/CRT-D, and the more severe the anxiety and depression, the worse the QoL [9–11]. A systematic review published in 2022 revealed that anxiety and depression are associated with an increased risk of mortality among patients with an ICD, although these psychological factors do not have a direct correlation with the incidence of ICD shocks [12]. Collectively, these findings highlight the significant impact of psychosocial factors on post-ICD implantation prognosis.

Social support means the perceived availability of resources including information and emotional or instrumental aids that can be obtained from individuals’ social network [13], which is another important psychosocial factor that impacts on adverse cardiac events. Among patients with coronary heart disease (CHD), a lack of social support correlates with higher cardiovascular mortality rates [14–17]. Hypertensive patients with low social support also face increased cardiovascular mortality, as shown in a 9-year cohort study [18]. Few studies have examined the impact of social support on outcomes after ICD/CRT-D implantation. A 1991 study found that half of ICD recipients experienced mental health disorders, often linked to inadequate social support [19]. A 2018 longitudinal study demonstrated that patients and partners who perceived higher social support pre-ICD implantation saw greater reductions in anxiety and depression symptoms [20]. Additionally, research involving ICD patients has shown that those who perceived better social support exhibited a more marked improvement in hemodynamic responses following mental stress [21]. There have been limited studies on social support and adverse cardiovascular events after ICD/CRT-D implantation so far.

The aim of this study was to investigate the longitudinal association between social support and adverse cardiac events during one-year follow-up after ICD/CRT-D implantation. To our best knowledge, this is the first such study conducted on ICD/CRT-D recipients. Given established evidence that anxiety/depression relate to worse cardiac prognosis [22, 23], we assessed them as potential confounders of social support effects. In addition, the association between anxiety or depression and cardiac events after implantation was also studied.

## Methods

### Study design

This was a hospital-based longitudinal study conducted at the First Affiliated Hospital of Kunming Medical University, China. Eligible patients undergoing first-time ICD/CRT-D implantation were consecutively enrolled from January 2020 to April 2023. The study protocol adhered to the ethical standards of the 1964 Declaration of Helsinki and its later amendments, with approval from the Ethics Committee of the First Affiliated Hospital of Kunming Medical University. All participants provided written informed consent prior to enrollment.

Data collection included two phases:

- Baseline assessment: Demographic characteristics, clinical data, and psychosocial metrics (social support, anxiety, depression) were collected within 72 h before ICD/CRT-D implantation.
- Follow-up: Participants were followed up at 1, 3, 6, and 12 months post-implantation via outpatient visits, inpatient records, or telephone interviews. At each follow-up, psychosocial metrics (anxiety, depression, social support) were re-evaluated, and cardiac events were documented. The primary objective was to explore the longitudinal association between social support, anxiety, depression, and adverse cardiac events over the 1-year follow-up period.

### Study participants

Participants were recruited from the Cardiology Department and Geriatric Cardiology Department of the First Affiliated Hospital of Kunming Medical University. The study cohort comprised a consecutively enrolled sample of 101 eligible patients who underwent first-time ICD/CRT-D implantation between January 2020 and April 2023.

#### Inclusion criteria

Patients were included if they met all the following conditions: (1) Met the ICD implantation criteria outlined in the 2017 AHA/ACC/HRS Guideline for Management of Patients with Ventricular Arrhythmias and the Prevention of Sudden Cardiac Death [24]; (2) Underwent first-time ICD/CRT-D implantation (no prior history of cardiac device implantation); (3) Received guideline-directed medical therapy during follow-up, defined as adherence to the 2017 AHA/ACC/HRS guidelines [24] (e.g., use of β-blockers, ACEI/ARB for heart failure, and antiarrhythmic drugs as recommended) with regular follow-up visits scheduled every 1–3 months and documented treatment adjustments (e.g., dose optimization) based on clinical status; (4) Had normal cognitive function, defined as a Mini-Mental State Examination (MMSE) score ≥ 24 for participants with ≥ 7 years of education or ≥ 20 for those with ≤ 6 years of education, a cutoff widely used in Chinese clinical research to indicate no significant cognitive impairment [25]; (5) Had a stable mental state, with no history of diagnosed mental disorders (e.g., schizophrenia, bipolar disorder) as confirmed by medical records; (6) Were able to read and understand the study questionnaires independently or with assistance (e.g., caregiver support for visual impairment).

#### Exclusion criteria

Patients were excluded for any of the following reasons: prior history of ICD/CRT-D implantation; the presence of severe comorbidities that could confound outcomes or prognosis, specifically end-stage liver disease (Child-Pugh class C [26]), end-stage renal disease (eGFR < 15 mL/min/1.73 m² [27]), active malignancy (requiring chemotherapy or radiotherapy within the preceding 6 months), or acute systemic infection requiring intravenous antibiotics at the time of enrollment; current pregnancy or breastfeeding; or a life expectancy of less than one year as determined by the attending physician based on the severity of underlying conditions (e.g., end-stage heart failure or advanced malignancy).

### Collection of demographic and clinical data

Demographic and clinical data were collected from patients’ medical records, including sex, age, body mass index (BMI), duration of heart disease (defined as the time since the initial diagnosis), New York Heart Association (NYHA) classification grading of cardiac function, previous medical history (coronary heart disease, hypertension, other cardiovascular diseases, diabetes mellitus, cerebrovascular diseases, etc.), history of smoking and alcohol consumption, family medical history, blood biochemical indicators, electrocardiogram, echocardiogram, and medication use.

### Anxiety and depression assessment

The Generalized Anxiety Disorder-7 (GAD-7) and the Patient Health Questionnaire-9 (PHQ-9) were used to evaluate anxiety and depression, respectively. The GAD-7, a simple and effective self-assessment tool for anxiety, consists of 7 items with a score range of 0–21, where higher scores indicate more severe anxiety symptoms [28]. The Chinese version of GAD-7 has shown good reliability and validity in Chinese populations [29]. The PHQ-9, with 9 items and a score range of 0–27, is a brief self-rated scale designed to assess depression symptoms, with higher scores indicating greater levels of depression [30]. The Chinese version of PHQ-9 has been validated with satisfactory reliability and validity [31]. In this study, the Cronbach’s alpha coefficients were 0.821 for GAD-7 and 0.832 for PHQ-9. Anxiety and depression levels were categorized into three groups based on validated cutoffs for the GAD-7 and PHQ-9 scales [28, 30]:

- No anxiety/depression: Scores < 5 (indicating an absence of clinically significant anxiety or depressive symptoms);
- Mild anxiety/depression: Scores 5–9 (reflecting mild, subclinical symptoms);
- Moderate-to-severe anxiety/depression: Scores ≥ 10 (indicating clinically relevant moderate to severe symptoms).

### Social support assessment

Social support was assessed by the Social Support Rating Scale (SSRS), which was developed for the Chinese population by Xiao [32], and this instrument has been validated by Liu et al. to have favorable reliability and validity [33]. The SSRS consists of 10 items, of which 4 items are for subjective support, 3 items are for objective support, and 3 items are for support utilization. It can be utilized to assess the level of social support over the past year. The total score of SSRS ranges from 12 to 66, and a higher score denotes higher level of social support [32]. Based on the original SSRS classification criteria for Chinese populations [32], participants were categorized into low-level support (≤ 19), medium-level support (20–29), and high-level support (≥ 30). However, since no participants scored ≤ 19 in our cohort, we dichotomized groups as low (20–29, equivalent to ‘medium’ in the original classification, reflecting relative lower support in our cohort) and high (≥ 30) support. In this study, the Cronbach’s alpha coefficient of the SSRS was 0.883, and the coefficients for the 3 dimensions of the SSRS were 0.831, 0.820, and 0.835, respectively.

### Definition of endpoint events

The primary composite endpoint was time to first occurrence of all-cause mortality or appropriate ICD/CRT-D shock (confirmed by intracardiac electrogram review for ventricular arrhythmia termination). The secondary composite endpoint was time to first occurrence of the primary endpoint event, heart failure hospitalization, or acute coronary syndrome (ACS). This hierarchical composite endpoint structure was employed to prioritize device-specific outcomes while concurrently capturing broader adverse cardiac events, a design that mitigates competing risk bias and is consistent with standard practices in cardiology trials [34–36].

All endpoints underwent blinded review by an independent clinical events committee. The time to event was calculated from device implantation to the earliest qualifying event.

### Statistical analysis

Given the single-center design and the consecutive enrollment of a convenience sample in this observational study, a formal a priori sample size calculation was not performed. This limitation is acknowledged in the Discussion. Post-hoc power analysis (PASS 2023, NCSS LLC) demonstrated that with 42 secondary endpoint events and an adjusted HR of 0.344 for social support, the study achieved 85% power (α = 0.05, two-sided) to detect this effect size. For anxiety/depression-time interactions (HR ≈ 1.001), power was 65–67% due to smaller effects. Notably, the power for the primary finding exceeded the 80% threshold for observational studies.

All statistical analyses were performed using Stata 17.0 software (Stata Corp, College Station, TX, USA). Continuous variables conforming to a normal distribution were presented as the mean ± standard deviation (SD). When the normal distribution assumption was violated, these variables were described using the median and interquartile range. Categorical variables were characterized by frequency and percentage (%).

Regarding social support, since no participants had scores of ≤ 19 on the Social Support Rating Scale (SSRS), we categorized social support into two groups based on baseline SSRS scores. Specifically, the low support group was defined as those with SSRS scores ranging from 20 to 29, while the high support group consisted of individuals with SSRS scores of ≥ 30. To compare the baseline characteristics between different social support groups, we employed the chi - square test for categorical variables. For normally distributed continuous variables, the independent samples t - test was utilized. When continuous variables did not follow a normal distribution, a natural logarithm transformation was applied to approximate normality before conducting the t - test.

Given the repeated measurements at five time points (baseline, 1, 3, 6, and 12 months), generalized estimation equations (GEEs) were used to analyze the temporal changes in anxiety, depression, and social support scores. GEEs are suitable for handling longitudinal data with correlated observations, enabling us to account for the within - subject correlation effectively.

Kaplan - Meier survival curves were constructed to evaluate the one - year event - free survival (EFS) rates across groups stratified by baseline levels of social support, anxiety, and depression. Group differences in survival curves were compared using log - rank tests. Additionally, Cox proportional hazards models were employed to assess the impact of baseline anxiety, depression, and social support on the occurrence of endpoint events, after adjusting for potential covariates. To further confirm the reliability of our findings regarding the role of core variables such as social support and to check the consistency of results across different modeling approaches, we simultaneously applied the Weibull models for analysis to verify whether the effects of these core variables, including social support, were consistent with those in the Cox proportional models.

For covariate selection, a two-stage process was adopted. First, variables showing a significant association with event-free survival (EFS) in univariate Kaplan-Meier analyses (log-rank test *p* < 0.05) were retained. Events were defined as the secondary composite endpoints (all-cause mortality, appropriate ICD shocks, heart failure hospitalization, or ACS). Second, demographic variables (age, sex) and clinical indicators (New York Heart Association [NYHA] class, left ventricular ejection fraction [LVEF], left ventricular end diastolic dimension [LVEDD]) were included in multivariable models based on their established prognostic value in cardiovascular research [37–40], irrespective of their univariate significance. Collinearity between variables was assessed using variance inflation factors, Pearson chi square test, or Spearman correlation analysis. This approach balanced data-driven selection with prior evidence to minimize overfitting.

Finally, to further explore how the effects of anxiety, depression, and social support on endpoint events varied over time, time - dependent Cox models were developed. To verify the results of the time - dependent Cox proportional hazards model and enhance the robustness of our findings, we employed a mixed - effects logistic regression model for a secondary analysis of the time - varying impact of social support on composite endpoint. This additional analysis provides a complementary perspective, helping to ensure the stability and generalizability of our conclusions.

All statistical analyses were conducted with a two-sided alpha level of 0.05 for significance. The hierarchical structure of the endpoints was explicitly considered in their interpretation: the primary endpoint provided the most specific test of device efficacy, while the secondary endpoint represented the overall adverse cardiovascular events.

## Results

### General information of participants

Among 133 consecutively screened patients, 32 were excluded (28 [87.5%] due to financial constraints preventing device affordability, 4 [12.5%] due to cognitive impairment). Thus, 101 participants were included in the final analysis. The baseline characteristics of the study cohort were summarized in Table 1.

**Table 1:** General characteristics of study participants

Category	Value
Baseline Diagnosis	
Ischemic heart disease	39 (38.6%)
Dilated heart disease	35 (34.7%)
Hypertensive heart disease	14 (13.8%)
Alcoholic heart disease	6 (5.9%)
Arrhythmogenic RV cardiomyopathy	3 (3.0%)
Valve disease	2 (2.0%)
Myocardial amyloidosis	1 (1.0%)
Hyperthyroidism cardiomyopathy	1 (1.0%)
**Demographics**	
Male / Female	77 (76.2%) / 24 (23.8%)
Age (years)	61.79 ± 11.54 (mean ± SD)
**Follow-up**	
Median follow-up duration	281 days
**Composite endpoints**	42 (41.58%)
All-cause death	8 (19.0% of events)
ICD/CRT-D appropriate shock	18 (42.9% of events)
Heart failure hospitalization	16 (38.1% of events)
ACS	0 (0% of events)
**Median time to events**	189 days

RV: Right ventricular; SD: Standard deviation; ICD: Implantable cardioverter-defibrillator; CRT-D: Cardiac resynchronization therapy devices with defibrillators; ACS: Acute coronary syndrome

### Baseline data among patients with two levels of social support

Based on their social support at baseline, 101 patients were divided into two groups: 39 with low support and 62 with high. Demographic and clinical data between these groups are presented in Supplemental Table 1. The high social support group was younger (mean age 59.9 vs. 64.9 years, *p* = 0.034), had higher serum albumin levels (39.2 vs. 37.2 g/L, *p* = 0.009), and better creatinine clearance (70.6 vs. 58.3 mL/min, *p* = 0.012) compared to the low support group. In addition, individuals with high social support had a higher prevalence of ICD implantation for primary prevention (62.90% vs. 25.64%, *p* < 0.0001). There were no significant differences in other factors (*p* > 0.05).

### Scores of anxiety, depression, and social support over time

Mean scores for anxiety, depression, and social support all exhibited an upward trend over the one-year follow-up. GEE analysis confirmed a statistically significant increase in anxiety scores (regression coefficient = 0.33, *p* < 0.001). In contrast, the increases in depression scores (regression coefficient = 0.15, *p* = 0.156) and social support scores (regression coefficient = 0.54, *p* = 0.140) were not statistically significant.

### Screening results for covariates

Kaplan-Meier curves of event-free survival (EFS) were plotted based on stratification by baseline demographic and clinical data. The corresponding log-rank test results were then summarized in Supplemental Table 2. Three variables demonstrated statistically significant differences in EFS (all *p* < 0.05): creatinine clearance rate (*p* < 0.0001), history of antiarrhythmic drug use (*p* = 0.0025), and indication for ICD implantation (*p* < 0.0001). To assess potential collinearity among these variables, Pearson’s chi-square tests were performed. The results revealed strong associations between ICD implantation indication and both creatinine clearance rate (χ² = 17.77, *p* < 0.0001) and antiarrhythmic drug use (χ² = 5.53, *p* = 0.019). Specifically, patients receiving ICDs for secondary prevention exhibited lower creatinine clearance and a higher prevalence of antiarrhythmic drug use compared to those in the primary prevention group. To avoid collinearity in the Cox models, only ICD implantation indication was retained as a covariate. Additionally, variables with established clinical relevance to post-MI cardiovascular events—age, sex, LVEF, LVEDD, and NYHA class—were considered for inclusion. While these factors did not reach statistical significance in log-rank tests (*p* ≥ 0.05), their inclusion was justified by prior evidence linking them to adverse cardiac outcomes. However, Spearman correlation analysis identified a moderate negative association between LVEF and NYHA class (ρ = -0.47, *p* < 0.001), as well as a moderate positive association between LVEDD and NYHA class (ρ = 0.46, *p* = 0.013). Given that NYHA class integrates both symptomatic and functional cardiac status, it was prioritized over LVEF or LVEDD to comprehensively capture disease severity and avoid redundancy. Finally, 4 covariates, including age, sex, NYHA class, and indication for ICD implantation, were included in the subsequent Cox proportional models.

### Impact of anxiety and depression on the incidence of endpoints in patients post ICD/CRT-D implantation

We categorized participants based on baseline anxiety and depression scores:

49 cases (48.5%) had GAD-7 scores < 5, 45 (44.6%) scored 5–9, and 7 (6.9%) had scores ≥ 10; for PHQ-9, 53 cases (52.5%) had scores < 5, 38 (37.6%) scored 5–9, and 10 (9.9%) had scores ≥ 10. The one-year event-free survival (EFS) stratified by these scores is shown in Supplemental Figs. 1 and 2. For the primary endpoint—first occurrence of all-cause mortality or ICD/CRT-D appropriate shock—neither anxiety (χ² = 1.13, *p* = 0.568) nor depression (χ² = 0.48, *p* = 0.787) showed significant effects. Similarly, for the secondary endpoints, which included primary endpoint components, heart failure hospitalization, and ACS, log-rank tests revealed no significant EFS differences across anxiety (χ² = 2.15, *p* = 0.342) or depression groups (χ² = 0.14, *p* = 0.934).

Tables 2 and 3 present Cox model results for secondary endpoints, while Supplemental Tables 3 and 4 detail those for primary endpoints. Notably, baseline anxiety and depression scores had no significant impact on the primary composite endpoints, and no interaction effects between these factors and time were observed for the primary endpoints. When delving into model-specific analyses, Table 2 shows that in multivariable survival analyses, anxiety scores trended towards increased risk in both Cox proportional hazards and Weibull models (adjusted HRs ≈ 1.1, all *p* > 0.10), yet this effect was non-significant. However, time-dependent Cox models uncovered a significant anxiety-time interaction (*p* < 0.001), suggesting that the risk associated with anxiety accumulates over the five follow-up periods (from baseline to 12 months). Similarly, Table 3 demonstrates that depression scores had no significant links to endpoints in standard Cox proportional hazards and Weibull models (hazard ratios around 1, *p* > 0.1). Although the main effect of depression scores remained non-significant in time-dependent Cox models, a significant depression-time interaction emerged (*p* < 0.001), indicating a time-related rise in depression-associated risk.

**Table 2:** Association of anxiety with secondary composite endpoints across models

Model type	Unadjusted		Adjusted	
	HR(95% CI)	*p*-value	HR(95% CI)	*p*-value
**Cox proportional hazards models**				
Anxiety score	1.074(0.978–1.179)	0.134	1.060(0.956–1.174)	0.270
**Weibull models**				
Anxiety score	1.074(0.980–1.177)	0.129	1.057(0.953–1.172)	0.295
**Time-dependent Cox proportional risk models**				
Anxiety score (main)	1.001(0.925–1.082)	0.986	0.971(0.895–1.054)	0.479
Anxiety score × Time	1.001(1.000-1.001)	< 0.001*	1.001(1.000-1.001)	< 0.001*

* p < 0.05

Abbreviations: *HR* Hazard ratio, *CI* Confidence interval

Notes:

1. Cox proportional hazards models

• Unadjusted: No covariates

• Adjusted: Adjusted for age, sex, NYHA class, and indication for ICD implantation

2. Weibull model

• Shape parameter p = 0.976, 95% CI (0.748–1.274) across adjusted model.• Adjustment strategies correspond to the column headers, as defined in Note 1

3. Time-dependent Cox proportional risk models

• Interaction term “Anxiety score × Time” tests the heterogeneity of the anxiety effect across 5 follow-up periods (baseline, 1-month, 3-month, 6-month, and 12-month)

• Adjustment strategies correspond to the column headers, as defined in Note 1

**Table 3:** Association of depression with secondary composite endpoints across models

Model type	Unadjusted		Adjusted	
	HR(95% CI)	*p*-value	HR(95% CI)	*p*-value
**Cox proportional hazards models**				
Depression score	1.010(0.902–1.130)	0.865	0.987(0.889–1.096)	0.812
**Weibull models**				
Depression score	1.010(0.906–1.125)	0.860	0.984(0.890–1.087)	0.747
**Time-dependent Cox proportional risk models**				
Depression score (main)	0.966(0.902–1.035)	0.325	0.948(0.887–1.013)	0.117
Depression score × Time	1.001(1.000-1.001)	< 0.001*	1.001(1.000-1.001)	< 0.001*

* p < 0.05

Abbreviations: *HR* Hazard ratio, *CI*  Confidence interval

Notes:

1. Cox proportional hazards models

• Unadjusted: No covariates

• Adjusted: Adjusted for age, sex, NYHA class, and indication for ICD implantation

2. Weibull model• Shape parameter p = 0.982, 95% CI (0.753–1.280) across adjusted model• Adjustment strategies correspond to the column headers, as defined in Note 1

3. Time-dependent Cox proportional risk models

• Interaction term “Depression score × Time” tests the heterogeneity of the depression effect across 5 follow-up periods (baseline, 1-month, 3-month, 6-month, and 12-month)

• Adjustment strategies correspond to the column headers, as defined in Note 1

### Impacts of social support on the incidence of endpoints in patients post ICD/CRT-D implantation

**Fig. 1:**
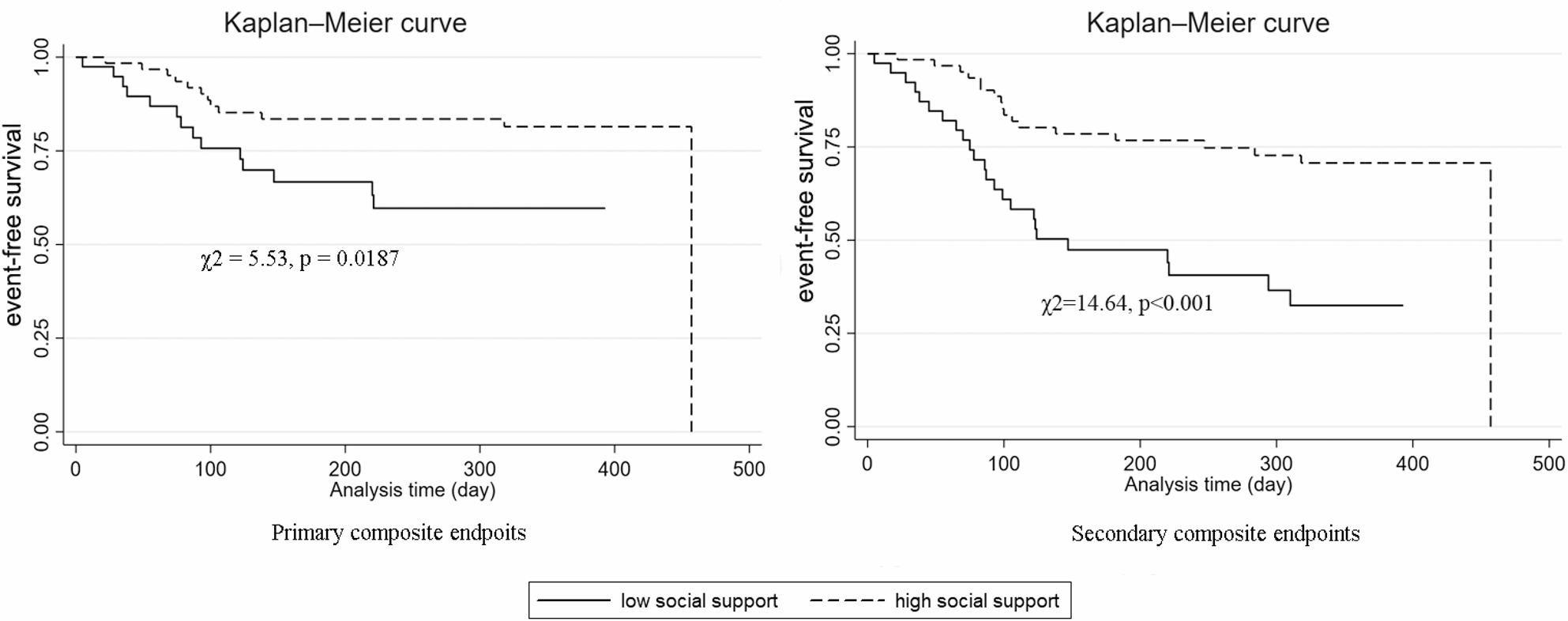
The event-free survival of patients by social support levels during a one-year follow-up period

Figure 1 illustrates the event-free survival (EFS) of patients stratified by baseline social support levels during the one-year follow-up period. The log-rank test results showed significant differences in EFS. For the primary endpoints, the test yielded a χ² value of 5.53 (*p* = 0.0187), while for the secondary endpoints, the χ² value was 14.64 (*p* < 0.001), indicating distinct survival patterns between the two social support groups.

We further explored the associations between social support and primary/secondary composite endpoints using multiple models. As presented in Supplemental Table 5, unadjusted analyses demonstrated that high social support was significantly associated with a reduced risk of primary endpoints. However, this significance diminished after adjusting for various covariates. In time-dependent Cox models, the main effect of high social support still significantly lowered the risk of primary endpoints, but the social support - time interaction had no significant impact.

For secondary endpoints, as detailed in Table 4, both unadjusted Cox and Weibull models indicated that high social support substantially decreased the risk. Moreover, this significant association remained robust after partial and full adjustment for relevant factors. In time-dependent Cox models, high social support exerted a significant protective main effect, and the “High social support × Time” interaction was significant, suggesting that the protective association of social support gradually weakened over time yet remained statistically significant.

To further validate the results of the time-dependent Cox proportional hazards model, we adopted an alternative approach—the mixed-effects logistic regression model. After adjusting for age, sex, NYHA class, indication for ICD implantation, and scores of anxiety and depression, the analysis showed that social support scores over time were negatively associated with secondary endpoint events during the one-year follow-up. The regression coefficient was − 0.061 (95% CI: -0.106 to -0.017; *p* = 0.007), which was consistent with the findings from the time-dependent Cox proportional hazards model, reinforcing the reliability of our results.

**Table 4:** Association of social support with secondary composite endpoints across models

Model type	Unadjusted		Partially adjusted		Fully adjusted	
	HR(95% CI)	*p*-value	HR(95% CI)	*p*-value	HR(95% CI)	*p*-value
**Cox proportional hazards models**						
High social support	0.314(0.168–0.588)	< 0.001*	0.374(0.188–0.745)	0.005*	0.344(0.172–0.688)	0.003*
**Weibull models**						
High social support	0.309(0.167–0.571)	< 0.001*	0.351(0.176-0.700)	0.003*	0.322(0.161–0.647)	0.001*
**Time-dependent Cox proportional risk models**						
High social support (main)	0.308(0.188–0.503)	< 0.001*	0.375(0.226–0.621)	< 0.001*	0.366(0.213–0.629)	< 0.001*
High social support × Time	0.990(0.982–0.997)	0.005*	0.991(0.984–0.998)	0.013*	0.991(0.983–0.999)	0.009*

* p < 0.05

Abbreviations: *HR* Hazard ratio, *CI* Confidence interval

Notes:

1. Cox proportional hazards models

• Unadjusted: No covariates

• Partially adjusted: Adjusted for age, sex, NYHA class, and indication for ICD implantation

• Fully adjusted: Partially adjusted model with additional adjustment for anxiety and depression scores

2. Weibull model

• Shape parameter p =1.048, 95% CI (0.806–1.363) across fully adjusted model

• Adjustment strategies correspond to the column headers, as defined in Note 1

3. Time-dependent Cox proportional risk models

• The interaction term "High social support × Time" tests the heterogeneity of the social support effect across the 5 follow-up periods (baseline, 1-month, 3-month, 6-month, and 12-month)

• Adjustment strategies correspond to the column headers, as defined in Note 1

## Discussion

Sudden cardiac death remains a significant public health challenge worldwide, necessitating strategies to mitigate its risk. This longitudinal study provides novel evidence on the protective role of psychosocial factors in a high-risk population, demonstrating that higher baseline social support is associated with a substantially reduced burden of overall cardiovascular morbidity among patients in the first year after ICD/CRT-D implantation. Our findings align with prior research on psychosocial determinants of cardiovascular outcomes but extend these insights to the understudied population of ICD/CRT-D recipients. Specifically, adjusted Cox models revealed that high social support correlated with a pronounced 65.6% risk reduction in the secondary composite endpoint (fully adjusted HR = 0.344, *p* = 0.003), which we specifically designed to capture the totality of significant adverse cardiovascular events, including heart failure hospitalization in addition to device-specific events (all-cause death and appropriate shocks). While a protective association was also observed for the primary endpoint (focused strictly on mortality and arrhythmias treated by the device), it was the association with this broader measure of clinical disease burden that proved most robust. This protective effect, though attenuated over time (HR = 0.991 per unit time, *p* = 0.009), remained statistically significant throughout follow-up.

The magnitude of this effect underscores the profound impact psychosocial factors can have on the clinical course of ICD/CRT-D recipients. The attenuated association between social support and the primary endpoint is consistent with the possibility that its putative benefits—such as better medication adherence, coping strategies, or hemodynamic profiles—are more strongly linked to a reduction in pathophysiological processes leading to heart failure exacerbation.

This research also revealed dynamic associations for anxiety and depression. Although their baseline levels showed no significant independent association with risk, a time-dependent escalation in risk was observed. This aligns with the fact that nearly 20% of individuals experience anxiety or depression post-ICD implantation [41], and our repeated measures documented a general upward trend in these scores over time. While a prior systematic review found no link between baseline psychological distress and ICD shocks [12], our longitudinal assessment suggests that worsening anxiety and depression over time may be associated with event incidence—a hypothesis requiring confirmation in larger studies.

Our findings indicate that the protective effect of social support gradually weakens over time, potentially influenced by increased levels of anxiety and depression. Given the inverse association between the severity of anxiety or depression and the level of social support [42–45], this interpretation is consistent with known biological mechanisms. However, during the follow-up period, we did not find a downward trend in the social support score. In addition, the inverse association between social support and endpoint events persisted after adjusting for anxiety and depression scores throughout the first-year follow-up, despite some attenuation over time. This suggests that the protective role of social support exists independently and is not dependent on anxiety and depression. Other pathways, such as behavioral processes encompassing health-promoting behaviors and adherence to medical prescriptions, could mediate the protective effect of social support [46].

We incorporated established prognostic markers in our analysis, including structural markers (left ventricular end-diastolic diameter [LVEDD]), electrophysiological parameters (QT dispersion [QTd], QRS duration), and functional indices (NYHA class, left ventricular ejection fraction [LVEF]). Prior studies have consistently linked left ventricular enlargement to poor prognosis [40, 47, 48], while prolonged QTd and QRS duration correlate with heightened arrhythmic risk and worse outcomes in heart failure [49–52]. Despite including these potentially crucial variables in our analysis, we found no significant differences in event-free survival among different social support groups, as detailed in Supplemental Table 2. Notably, patients receiving ICDs for secondary prevention (i.e., prior life-threatening arrhythmia) faced a higher risk of adverse events, likely reflecting their advanced cardiac pathology. Importantly, even after adjusting for confounding factors such as age, sex, NYHA class, and implantation indication, social support remained associated with reduced event risk (Table 4). These findings underscore low social support as an independent risk factor in ICD/CRT-D recipients.

To date, limited research has examined the impact of social support on hard clinical events in ICD/CRT-D recipients. As has been noted, lack of social support relates to mental disorders in ICD recipients and their partners [19, 20], and negatively influences their quality of life [53]. Few studies have explored the association between social support and cardiac events in ICD/CRT-D recipients. This study found that higher social support is associated with fewer cardiac events, but this protective association diminishes over time, suggesting a need for ongoing psychosocial support. Based on these findings, future interventions tailored to the Chinese context could be explored, encompassing: (1) hospital-based peer support led by cardiology nurses; (2) community health worker-led home visits to reinforce adherence and family engagement; and (3) digital platforms (e.g., WeChat groups) for education and support. These proposed strategies leverage existing resources and align with cultural norms of family-centric care, ensuring feasibility. Previous strategies such as brief routine communications between healthcare providers and patients [54, 55], support group recommendations [56], computerized psychosocial care [57], and web-based interventions [58] have also shown promise in enhancing ICD recipients’ well-being, though a recent review noted inconsistent effects of social support interventions on heart disease-related mortality, highlighting the need for further research with clearer theorization [59].

This study has several limitations. First, the single-center design may restrict the generalizability of findings to other regions within China. Furthermore, differences in healthcare systems (e.g., ICD reimbursement policies) and clinical practices (e.g., device selection criteria) between China and other countries may limit extrapolation to international populations. Second, the modest sample size (*n* = 101), a consequence of the single-center observational design in which a formal a priori sample size calculation was not performed, may have reduced the power to detect smaller effect sizes, particularly for the associations involving anxiety and depression. To address this, we validated key findings using both Cox and mixed-effects models, ensuring robustness across analytical approaches. This was complemented by a post-hoc power analysis, which indicated adequate power for the primary finding regarding social support. Third, the exclusion of 28 patients (21% of screened individuals) due to financial constraints may have introduced socio-economic selection bias. Since socioeconomic status is closely linked to both device accessibility and social support, this may limit the generalizability of our findings, particularly in settings with variable healthcare coverage or to patient populations with significant financial barriers. Fourth, despite adjusting for key covariates in our models, residual confounding may persist due to clinically relevant baseline differences between social support groups (e.g., younger age, better renal function, and a higher proportion of primary prevention ICD indication in the high-support group). Although these factors were included in our multivariable models, unmeasured or imperfectly measured confounders could still influence the observed associations. Fifth, social support, anxiety, and depression were assessed via self-report scales, which may introduce measurement bias (e.g., social desirability bias). Sixth, mixed data collection methods (outpatient visits, inpatient follow-ups, and telephone calls) may introduce bias, such as variability in data granularity or recall bias in psychosocial metric reporting. To mitigate this, we standardized data collection forms, trained staff to administer assessments consistently, and verified critical endpoints via objective records; residual bias may nonetheless persist. Despite these limitations, our study provides novel insights into the prognostic role of social support in ICD/CRT-D recipients.

## Conclusions

Our study demonstrates that higher social support is a significant, independent predictor of a reduced burden of cardiovascular events (65.6% risk reduction) in the first year after ICD/CRT-D implantation. This protective association, derived from our analysis of the secondary composite endpoint, highlights the potential for psychosocial interventions to improve comprehensive patient outcomes by targeting a broad range of adverse clinical events. Additionally, while baseline anxiety and depression were not independently associated with initial risk, their association with adverse outcomes appears to escalate over time, underscoring the need for sustained psychological monitoring and support.

## Supplementary Information

Supplementary Material 1.

Supplementary Material 2.

Supplementary Material 3.

Supplementary Material 4.

Supplementary Material 5.

Supplementary Material 6.

Supplementary Material 7.

Supplementary Material 8.

## Data Availability

Data and materials are provided within the manuscript or supplementary information files.
