# Supplementary material for: High social support is associated with reduced cardiac events in patients following ICD/CRT-D implantation: a one-year follow-up study in China
Source: BMC Psychol. 2025 Dec 30;14:133. doi: 10.1186/s40359-025-03912-5 (PMC12857033; doi:10.1186/s40359-025-03912-5)
Supplement: Supplementary file 1 — Supplementary Material 1. [file 40359_2025_3912_MOESM1_ESM.docx]

**Supplemental Table 1. Baseline data among patients with different levels of social support**

|  | Social support level | | t/χ² value | *P* value |
| --- | --- | --- | --- | --- |
|  | ≤29（n=39） | ≥30 (n=62) |  |  |
| Age (year) | 64.85±13.44 | 59.87±9.81 | 2.15 | 0.034* |
| Male [n,(%)] | 31（79.49） | 46（74.19） | 0.37 | 0.543 |
| BMI (kg/m^2^) | 22.79±3.28 | 23.92±3.85 | -1.52 | 0.131 |
| Duration of heart disease (Ln day) ^#^ | 6.50±1.55 | 6.40±1.72 | 0.31 | 0.760 |
| Hypertension [n,(%)] | 21（53.85） | 24（38.71） | 2.22 | 0.136 |
| CHD [n,(%)] | 15（38.46） | 24（38.71） | 0.00 | 0.980 |
| Diabetes [n,(%)] | 6（15.38） | 14（22.58） | 0.78 | 0.377 |
| Stroke [n,(%)] | 5（12.82） | 7（11.29） | 0.05 | 0.817 |
| Smoking at present [n,(%)] | 11（28.21） | 18（29.03） | 0.01 | 0.929 |
| Heavy drinker [n,(%)] † | 12（30.77） | 16（25.81） | 0.29 | 0.587 |
| Family history of CVD [n,(%)] | 6 (15.38) | 14 (22.58) | 0.78 | 0.377 |
| Blood biochemical indexes | | | | |
| ALB (g/L) | 37.15±3.22 | 39.15±3.95 | -2.66 | 0.009* |
| ALT (Ln IU/L) ^#^ | 3.56±0.87 | 3.44±0.78 | 0.70 | 0.485 |
| CCR (ml/min) | 58.33±25.82 | 70.56±21.90 | -2.55 | 0.012* |
| Electrocardiogram |  |  |  |  |
| QTd (ms) | 73.56±18.40 | 66.82±22.18 | 1.59 | 0.116 |
| QRS duration (ms) | 120.15±27.76 | 114.63±25.69 | 1.02 | 0.310 |
| Echocardiography |  |  |  |  |
| LVEF (%) | 46.29±11.78 | 45.00±15.28 | 0.45 | 0.657 |
| LVEDD (mm) | 63.10±13.89 | 62.21±13.05 | 0.33 | 0.745 |
| NYHA classification grading of cardiac function [n,(%)] | | | 0.89 | 0.827 |
| Grade I | 3（7.69） | 7（11.29） |  |  |
| Grade II | 12（30.77） | 20（32.26） |  |  |
| Grade III | 19（48.72） | 25（40.32） |  |  |
| Grade IV | 5（12.82） | 10（16.13） |  |  |
| Pacemaker type [n,(%)] | |  | 0.63 | 0.731 |
| Single chamber ICD | 9（47.06） | 16（25.81） |  |  |
| Dual chamber ICD | 22 (35.29) | 37 (59.68) |  |  |
| CRT-D | 8（17.65） | 9（14.52） |  |  |
| Indication for ICD implantation [n,(%)] | |  | 13.31 | <0.0001* |
| Primary prevention | 10 (25.64) | 39 (62.90) |  |  |
| Secondary prevention | 29 (74.36) | 23 (37.10) |  |  |
| Medication usage. |  |  |  |  |
| Beta-blocker [n,(%)] | 39（100.00） | 57（91.94） | 3.31 | 0.069 |
| MRA [n,(%)] | 33（84.62） | 54（87.10） | 0.12 | 0.725 |
| RASI [n,(%)] | 30（76.92） | 42（67.74） | 0.99 | 0.321 |
| SGLT-2i [n,(%)] | 21（53.85） | 34（54.84） | 0.01 | 0.922 |
| Digoxin [n,(%)] | 5（12.82） | 5（8.06） | 0.61 | 0.436 |
| Loop diuretic [n,(%)] | 32（82.05） | 46（74.19） | 0.84 | 0.359 |
| Anti-arrhythmic drugs [n,(%)] ‡ | 5（12.82） | 7（11.29） | 0.05 | 0.817 |
| Anxiety score | 4.59±2.09 | 5.18±3.04 | -1.06 | 0.292 |
| Depression score | 4.44±2.85 | 5.02±3.06 | -0.95 | 0.344 |

**p*＜0.05

# The data does not conform to a normal distribution and has been converted to its natural logarithm to conform to a normal distribution.

† Heavy drinker means the average daily alcohol intake exceeds 60 grams for men and 40 grams for women.

‡ Anti-arrhythmic drugs include amiodarone, mexiletine, propafenone, and dronedarone.

BMI: body mass index; Ln: natural logarithm; CHD: coronary heart disease; CVD: cardiovascular disease; ALB: albumin; ALT: alanine transaminase; CCR: creatinine clearance rate; MRA: mineralcorticoid receptor antagonist; RASI: renin-angiotensin system inhibitor; SGLT-2i: sodium-dependent glucose transporters-2 inhibitor; QTd: QT dispersion; LVEDD: left ventricular end diastolic diameter; LVEF: left Ventricular ejection fraction.
