# Supplementary material for: High social support is associated with reduced cardiac events in patients following ICD/CRT-D implantation: a one-year follow-up study in China
Source: BMC Psychol. 2025 Dec 30;14:133. doi: 10.1186/s40359-025-03912-5 (PMC12857033; doi:10.1186/s40359-025-03912-5)
Supplement: Supplementary file 2 — Supplementary Material 2. [file 40359_2025_3912_MOESM2_ESM.docx]

**Supplemental Table 2. Log-rank test results for event-free survival of potential risk factors**

|  | | Event-free group  (n=59) | Event group  (n=42) | Log-rank test (χ^2^ value) | *P* value |
| --- | --- | --- | --- | --- | --- |
| Age (years) | |  |  | 5.19 | 0.159 |
|  | 32-55 | 19(32.20%) | 9(21.43%) |  |  |
|  | 56-62 | 16(27.12%) | 8(19.05%) |  |  |
|  | 63-71 | 11(18.64%) | 15(35.71%) |  |  |
|  | 72-84 | 13(22.03%) | 10(23.81%) |  |  |
| Sex | |  |  | 0.03 | 0.852 |
|  | Male | 46(77.97%) | 31(73.81%) |  |  |
|  | Female | 13(22.03%) | 11(26.19%) |  |  |
| BMI（kg/m^2^） | |  |  | 0.99 | 0.610 |
|  | <24 | 35(59.32%) | 27(64.29%) |  |  |
|  | 24-28 | 14(23.73%) | 11(26.19%) |  |  |
|  | >28 | 10(16.95%) | 4(9.52%) |  |  |
| Duration of heart disease (Ln day) ^#^ | |  |  | 6.41 | 0.093 |
|  | 1.95-5.22 | 23(38.98%) | 7(16.67%) |  |  |
|  | 5.23-7.00 | 12(20.34%) | 11(26.19%) |  |  |
|  | 7.01-7.85 | 14(23.73%) | 12(28.57%) |  |  |
|  | 7.86-8.61 | 10(16.95%) | 12(28.57%) |  |  |
| Hypertension | |  |  | 0.89 | 0.347 |
|  | No | 35(59.32%) | 21(50.00%) |  |  |
|  | Yes | 24(40.68%) | 21(50.00%) |  |  |
| CHD | |  |  | 1.58 | 0.209 |
|  | No | 33(55.93%) | 29(69.05%) |  |  |
|  | Yes | 26(44.07%) | 13(30.95%) |  |  |
| Diabetes | |  |  | 1.41 | 0.234 |
|  | No | 45(76.27%) | 36(85.71%) |  |  |
|  | Yes | 14(23.73%) | 6(14.29%) |  |  |
| Stroke | |  |  | 0.13 | 0.721 |
|  | No | 51(86.44%) | 38(90.48%) |  |  |
|  | Yes | 8(13.56%) | 4(9.52%) |  |  |
| Family history of CVD | |  |  | 0.00 | 0.952 |
|  | No | 47(79.66%) | 34(80.95%) |  |  |
|  | Yes | 12(20.34%) | 8(19.05%) |  |  |
| Smoking at present | | |  | 0.10 | 0.752 |
|  | No | 41(69.49%) | 31(73.81%) |  |  |
|  | Yes | 18(30.51%) | 11(26.19%) |  |  |
| Heavy drinker † | |  |  | 0.18 | 0.673 |
|  | No | 42(71.19%) | 31(73.81%) |  |  |
|  | Yes | 17(28.81%) | 11(26.19%) |  |  |
| ALB（g/L） | |  |  | 3.64 | 0.303 |
|  | 30.50-36.30 | 13(22.03%) | 14(33.33%) |  |  |
|  | 36.31-38.35 | 11(18.64%) | 12(28.57%) |  |  |
|  | 38.36-41.20 | 17(28.81%) | 8(19.05%) |  |  |
|  | 41.21-47.60 | 18(30.51%) | 8(19.05%) |  |  |
| ALT (Ln IU/L) ^#^ | |  |  | 3.82 | 0.282 |
|  | 2.07-2.94 | 20(33.90%) | 7(16.67%) |  |  |
|  | 2.95-3.36 | 13(22.03%) | 12(28.57%) |  |  |
|  | 3.37-3.97 | 12(20.34%) | 13(30.95%) |  |  |
|  | 3.98-6.89 | 14(23.73%) | 10(23.81%) |  |  |
| CCR (ml/min) | |  |  | 34.12 | <0.0001* |
|  | >70 | 36(61.02%) | 5(11.90%) |  |  |
|  | 51-70 | 18(30.51%) | 20(47.62%) |  |  |
|  | 31-50 | 4(6.78%) | 12(28.57%) |  |  |
|  | <30 | 1(1.69%) | 5(11.90%) |  |  |
| QTd (ms) | |  |  | 2.41 | 0.121 |
|  | ＜80 | 42(71.19%) | 22(52.38%) |  |  |
|  | ≥80 | 17(28.81%) | 20(47.62%) |  |  |
| QRS duration (ms) | |  |  | 1.90 | 0.168 |
|  | ＜120 | 40(67.80%) | 21(50.00%) |  |  |
|  | ≥120 | 19(32.20%) | 21(50.00%) |  |  |
| LVEF (%) | |  |  | 3.70 | 0.157 |
|  | ＜40 | 17(28.81%) | 15(35.71%) |  |  |
|  | 40-49 | 14(23.73%) | 15(35.71%) |  |  |
|  | ≥50 | 28(47.46%) | 12(28.57%) |  |  |
| LVEDD(mm) | |  |  | 0.84 | 0.360 |
|  | ＜55 | 17(28.81%) | 9(21.43%) |  |  |
|  | ≥55 | 42(71.19%) | 33(78.57%) |  |  |
| NYHA classification grading of cardiac function | | | | 4.29 | 0.232 |
|  | Grade I | 5(8.47%) | 5(11.90%) |  |  |
|  | Grade II | 22(37.29%) | 10(23.81%) |  |  |
|  | Grade III | 26(44.07%) | 18(42.86%) |  |  |
|  | Grade IV | 6(10.17%) | 9(21.43%) |  |  |
| Beta-blocker | | |  | 0.96 | 0.328 |
|  | No | 4(6.78%) | 1(2.38%) |  |  |
|  | Yes | 55(93.22%) | 41(97.62%) |  |  |
| MRA | |  |  | 0.17 | 0.680 |
|  | No | 9(15.25%) | 5(11.90%) |  |  |
|  | Yes | 50(84.75%) | 37(88.10%) |  |  |
| RASI | |  |  | 0.09 | 0.759 |
|  | No | 17(28.81%) | 12(28.57%) |  |  |
|  | Yes | 42(71.19%) | 30(71.43%) |  |  |
| SGLT-2i | | |  | 0.05 | 0.824 |
|  | No | 26(44.07%) | 20(47.62%) |  |  |
|  | Yes | 33(55.93%) | 22(52.38%) |  |  |
| Loop diuretic | | |  | 0.23 | 0.635 |
|  | No | 15(25.42%) | 8(19.05%) |  |  |
|  | Yes | 44(74.58%) | 34(80.95%) |  |  |
| Digoxin | |  |  | 0.90 | 0.344 |
|  | No | 55(93.22%) | 36(85.71%) |  |  |
|  | Yes | 4(6.78%) | 6(14.29%) |  |  |
| Anti-arrhythmic drugs ‡ | | |  | 9.14 | 0.0025* |
|  | No | 55(93.22%) | 34(80.95%) |  |  |
|  | Yes | 4(6.78%) | 8(19.05%) |  |  |
| Pacemaker type | | |  | 1.03 | 0.598 |
|  | Single chamber ICD | 16(27.12%) | 9(21.43%) |  |  |
|  | Dual chamber ICD | 36(61.02%) | 23(54.76%) |  |  |
|  | CRT-D | 7(11.86%) | 10(23.81%) |  |  |
| Indication for ICD implantation | | |  | 17.31 | <0.0001* |
|  | Primary prevention | 38(64.41%) | 11(26.19%) |  |  |
|  | Secondary prevention | 21(35.59%) | 31(73.81%) |  |  |

Events were defined as all-cause mortality, appropriate ICD shocks, heart failure hospitalization, or acute coronary syndrome.

Categorical variables underwent direct Kaplan-Meier analysis, while continuous variables were grouped based on established grouping standards (BMI, CCR, QTd, QRS duration, LVEF, and LVEDD) or quartiles (age, duration of heart disease, ALT, and ALB) before Kaplan-Meier analysis.

**p*＜0.05.

# The data does not conform to a normal distribution and has been converted to its natural logarithm to conform to a normal distribution.

† Heavy drinker means the average daily alcohol intake exceeds 60 grams for men and 40 grams for women.

‡ Anti-arrhythmic drugs include amiodarone, mexiletine, propafenone, and dronedarone.

BMI: body mass index; CVD: cardiovascular disease; ALB: albumin; CHD: coronary heart disease; ALT: alanine transaminase; CCR: creatinine clearance rate; MRA: mineralcorticoid receptor antagonist; RASI: renin-angiotensin system inhibitor; SGLT-2i: sodium-dependent glucose transporters-2 inhibitor; QTd: QT dispersion; LVEDD: left ventricular end diastolic diameter; LVEF: left Ventricular ejection fraction.
