# Supplementary material for: High social support is associated with reduced cardiac events in patients following ICD/CRT-D implantation: a one-year follow-up study in China
Source: BMC Psychol. 2025 Dec 30;14:133. doi: 10.1186/s40359-025-03912-5 (PMC12857033; doi:10.1186/s40359-025-03912-5)
Supplement: Supplementary file 3 — Supplementary Material 3. [file 40359_2025_3912_MOESM3_ESM.docx]

**Supplemental Table 3. Association of anxiety with primary composite endpoints across models**

| **Model type** | **Unadjusted** | | **Adjusted** | |
| --- | --- | --- | --- | --- |
|  | HR(95% CI) | *p*-value | HR(95% CI) | *p*-value |
| **Cox proportional hazards models** | | | | |
| Anxiety score | 1.005(0.872-1.158) | 0.950 | 0.975(0.848-1.120) | 0.721 |
| **Weibull models** | | | | |
| Anxiety score | 1.001(0.871-1.149) | 0.993 | 0.970(0.844-1.116) | 0.671 |
| **Time-dependent Cox proportional risk models** | | | | |
| Anxiety score (main) | 0.953(0.853-1.063) | 0.386 | 0.919(0.818-1.032) | 0.154 |
| Anxiety score × Time | 1.000(0.999-1.001) | 0.271 | 1.000(0.999-1.001) | 0.217 |

**p*<0.05

Abbreviations: HR = Hazard ratio; CI = Confidence interval.

Notes:

1. Cox proportional hazards models

- - Unadjusted: No covariates.
  - Adjusted: Adjusted for age, gender, NYHA class, and indication for ICD implantation.

2. Weibull model

- Shape parameter p = 0.839, 95% CI (0.593–1.188) across adjusted model.
- Adjustment strategy same as Cox models.

3. Time-dependent Cox proportional risk models

- Interaction term “Anxiety score × Time” tests the heterogeneity of the anxiety effect across 5 follow-up periods (baseline, 1-month, 3-month, 6-month, and 12-month).
- Adjustment strategy same as Cox models.
