# Supplementary material for: High social support is associated with reduced cardiac events in patients following ICD/CRT-D implantation: a one-year follow-up study in China
Source: BMC Psychol. 2025 Dec 30;14:133. doi: 10.1186/s40359-025-03912-5 (PMC12857033; doi:10.1186/s40359-025-03912-5)
Supplement: Supplementary file 4 — Supplementary Material 4. [file 40359_2025_3912_MOESM4_ESM.docx]

**Supplemental Table 4. Association of depression with primary composite endpoints across models**

| **Model type** | **Unadjusted** | | **Adjusted** | |
| --- | --- | --- | --- | --- |
|  | HR(95% CI) | *p*-value | HR(95% CI) | *p*-value |
| **Cox proportional hazards models** | | | | |
| Depression score | 1.046(0.908-1.204) | 0.533 | 1.008(0.881-1.154) | 0.909 |
| **Weibull models** | | | | |
| Depression score | 1.041(0.908-1.192) | 0.564 | 0.998(0.875-1.138) | 0.972 |
| **Time-dependent Cox proportional risk models** | | | | |
| Depression score (main) | 1.062(0.981-1.149) | 0.137 | 1.061(0.978-1.152) | 0.155 |
| Depression score × Time | 1.000(0.999-1.001) | 0.673 | 1.000(0.999-1.001) | 0.832 |

**p*<0.05

Abbreviations: HR = Hazard ratio; CI = Confidence interval.

Notes:

1. Cox proportional hazards models

- - Unadjusted: No covariates.
  - Adjusted: Adjusted for age, gender, NYHA class, and indication for ICD implantation.

2. Weibull model

- Shape parameter p = 0.836, 95% CI (0.590–1.183) across adjusted model.
- Adjustment strategy same as Cox models.

3. Time-dependent Cox proportional risk models

- Interaction term “Depression score × Time” tests the heterogeneity of the depression effect across 5 follow-up periods (baseline, 1-month, 3-month, 6-month, and 12-month).
- Adjustment strategy same as Cox models.
