# Supplementary material for: High social support is associated with reduced cardiac events in patients following ICD/CRT-D implantation: a one-year follow-up study in China
Source: BMC Psychol. 2025 Dec 30;14:133. doi: 10.1186/s40359-025-03912-5 (PMC12857033; doi:10.1186/s40359-025-03912-5)
Supplement: Supplementary file 5 — Supplementary Material 5. [file 40359_2025_3912_MOESM5_ESM.docx]

**Supplemental Table 5. Association of social support with primary composite endpoints across models**

| **Model type** | **Unadjusted** | | **Partially adjusted** | | **Fully adjusted** | |
| --- | --- | --- | --- | --- | --- | --- |
|  | HR(95% CI) | *p*-value | HR(95% CI) | *p*-value | HR(95% CI) | *p*-value |
| **Cox proportional hazards models** | | | | |  |  |
| High social support | 0.399(0.181-0.882) | 0.023* | 0.460(0.193-1.096) | 0.079 | 0.463(0.193-1.110) | 0.084 |
| **Weibull models** | | | | |  |  |
| High social support | 0.399(0.184-0.866) | 0.020* | 0.439(0.184-1.049) | 0.064 | 0.442(0.183-1.065) | 0.069 |
| **Time-dependent Cox proportional risk models** | | | | |  |  |
| High social support (main) | 0.392(0.150-0.538) | <0.001* | 0.447(0.228-0.639) | <0.001* | 0.428(0.195-0.520) | <0.001* |
| High social support × Time | 1.002(0.997-1.007) | 0.413 | 1.000(0.995-1.005) | 0.648 | 1.002(0.996-1.008) | 0.560 |

** p* < 0.05

Abbreviations: HR = Hazard ratio; CI = Confidence interval.

Notes:

1. Cox proportional hazards models

- - Unadjusted: No covariates.
  - Partially adjusted: Adjusted for age, gender, NYHA class, and indication for ICD implantation.
  - Fully adjusted: Partially adjusted + adjusted for anxiety and depression scores.

2. Weibull model

- Shape parameter p =0.863, 95% CI (0.611–1.220) across fully adjusted model.
- Adjustment strategy same as Cox models.

3. Time-dependent Cox proportional risk models

- Interaction term “High social support × Time” tests the heterogeneity of the social support effect across 5 follow-up periods (baseline, 1-month, 3-month, 6-month, and 12-month).
- Adjustment strategy same as Cox models.
