# Supplementary figures and images for: High social support is associated with reduced cardiac events in patients following ICD/CRT-D implantation: a one-year follow-up study in China
Source: BMC Psychol. 2025 Dec 30;14:133. doi: 10.1186/s40359-025-03912-5 (PMC12857033; doi:10.1186/s40359-025-03912-5)

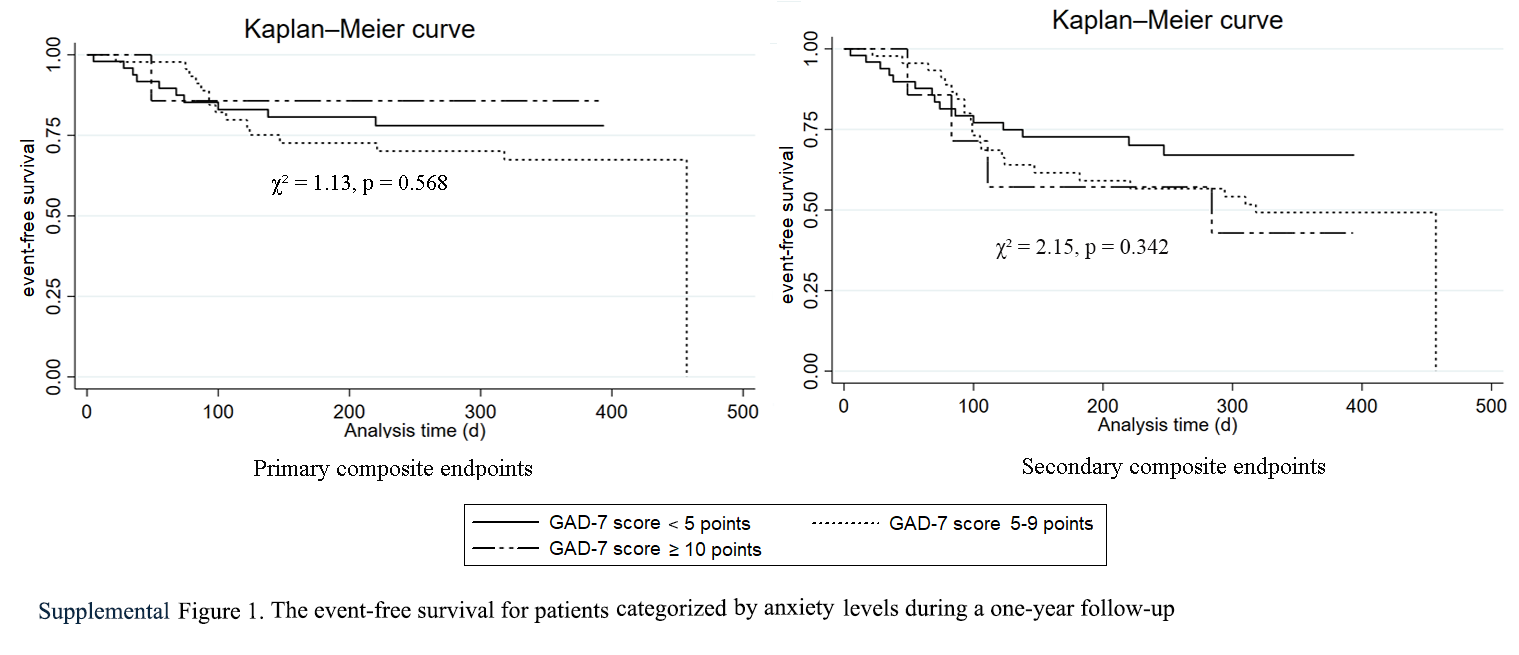

Supplement: Supplementary file 6 — Supplementary Material 6. [file 40359_2025_3912_MOESM6_ESM.tif]

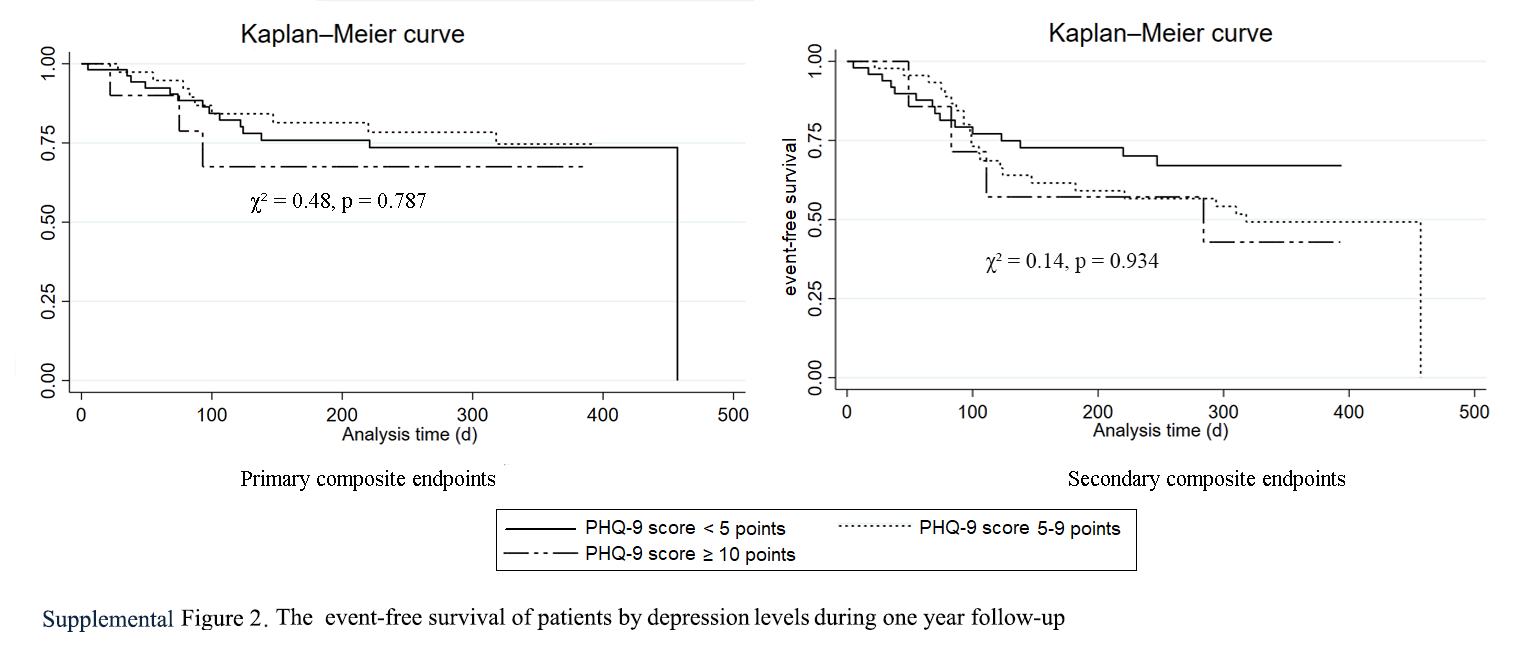

Supplement: Supplementary file 7 — Supplementary Material 7. [file 40359_2025_3912_MOESM7_ESM.tif]
