# Supplementary material for: High social support is associated with reduced cardiac events in patients following ICD/CRT-D implantation: a one-year follow-up study in China
Source: BMC Psychol. 2025 Dec 30;14:133. doi: 10.1186/s40359-025-03912-5 (PMC12857033; doi:10.1186/s40359-025-03912-5)
Supplement: Supplementary file 8 — Supplementary Material 8. [file 40359_2025_3912_MOESM8_ESM.pdf]

社会支持评定量表

Social Support Rating Scale

Gender: ______ Age: ______ years Occupation: ______ Marital status: ______

Date of completion: ______ month ______ day

The following questions aim to reflect the support you receive in society. Please answer according to your actual situation. Thank you for your cooperation.

1. How many close friends can you rely on for support and help? (Choose one)

(1) None

(2) 1

(3) 2

(4) 3 or more

2. Your living situation: (Choose one)

(1) Living alone

(2) Frequently moving, mostly staying with strangers

(3) Living with classmates, colleagues, or friends

(4) Living with family

3. Your relationship with neighbors: (Choose one)

(1) Never care about each other, only nodding acquaintances

(2) May show slight concern when facing difficulties

(3) Some neighbors care about you

(4) Most neighbors care about you

4. Your relationship with colleagues: (Choose one)

(1) Never care about each other, only nodding acquaintances

(2) May show slight concern when facing difficulties

(3) Some colleagues care about you

(4) Most colleagues care about you

5. Support and care received from family members (Mark "√" in the appropriate box)

	None	Seldom	Average	Full support
A. Spouse/ partner				
B. Parents				
C. Children				
D. Siblings				
E. Other members (e.g., sister-in-law)				

6. In the past, when you faced emergencies, the sources of financial support or practical help you received were:

(1) No sources at all

(2) The following sources (multiple choices allowed):

A. Spouse; B. Other family members; C. Friends; D. Relatives; E. Colleagues;

F. Workplace; G. Official or semi-official organizations (e.g., Communist Party, League, trade unions);

H. Non-official organizations (e.g., religious groups, social associations); I. Others (please specify): ______

7. In the past, when you faced emergencies, the sources of comfort and care you received were:

(1) No sources at all

(2) The following sources (multiple choices allowed):

A. Spouse; B. Other family members; C. Friends; D. Relatives; E. Colleagues;

F. Workplace; G. Official or semi-official organizations (e.g., Communist Party, League, trade unions);

H. Non-official organizations (e.g., religious groups, social associations); I. Others (please specify):

______

8. How do you vent your troubles? (Choose one)

(1) Never tell anyone

(2) Only tell 1-2 extremely close people

(3) Will talk about it if friends ask actively

(4) Take the initiative to talk about your troubles to get support and understanding

9. How do you seek help when in trouble? (Choose one)

(1) Rely only on yourself and refuse help from others

(2) Seldom ask others for help

(3) Sometimes ask others for help

(4) Often turn to family, relatives, friends, or organizations for help when in trouble

10. Regarding activities organized by groups (e.g., Party organizations, religious organizations, public welfare groups, student unions, etc.), you: (Choose one)

(1) Never participate

(2) Participate occasionally

(3) Participate frequently

(4) Take the initiative to participate and be actively involved

Total score: ______

Scoring Method for the Social Support Rating Scale

1. Items 1-4, 8-10: Each item has only one choice. Scores correspond to choices 1, 2, 3, 4 as 1, 2, 3, 4 points respectively.
2. Item 5: Scores are summed across items A, B, C, D, E. Each item is scored 1-4 points from "None" to "Full support".
3. Items 6 and 7: If the answer is "No sources at all", score 0 points. If answering "The following sources ", score 1 point for each source selected.

Analysis Method for the Social Support Rating Scale

1. Total score: Sum of scores from all 10 items.
2. Objective support score: Sum of scores from items 2, 6, and 7.
3. Subjective support score: Sum of scores from items 1, 3, 4, and 5.
4. Utilization of support: Scores from items 8, 9, and 10.

Note: This is an unofficial English translation of SSRS (unverified, produced through pre - and post translation), intended for international readers to understand and read.
